# Inflammatory Cytokines and Chemokines Are Synergistically Induced in a ROS-Dependent Manner by a Co-Culture of Corneal Epithelial Cells and Neutrophil-like Cells in the Presence of Particulate Matter

**DOI:** 10.3390/antiox13040467

**Published:** 2024-04-16

**Authors:** Zirui Zeng, Yasuhiro Yoshida, Duo Wang, Yuri Fujii, Mengyue Shen, Tatsuya Mimura, Yoshiya Tanaka

**Affiliations:** 1 The First Department of Internal Medicine, School of Medicine, University of Occupational and Environmental Health, Japan, 1-1 Iseigaoka, Yahatanishi-ku, Kitakyushu 807-8555, Japan; 2 Department of Immunology and Parasitology, School of Medicine, University of Occupational and Environmental Health, Japan, 1-1 Iseigaoka, Yahatanishi-ku, Kitakyushu 807-8555, Japan; z201078@info.uoeh-u.ac.jp (Y.F.);; 3 Department of Radiobiology and Hygiene Management, Institute of Industrial Ecological Sciences, University of Occupational and Environmental Health, Japan, 1-1 Iseigaoka, Yahatanishi-ku, Kitakyushu 807-8555, Japan; 4 Department of Medical Teaching, West China Center of Medical Sciences of Sichuan University, Chengdu 610041, China; 5 Department of Ophthalmology, Teikyo University School of Medicine, Tokyo 173-0003, Japan

**Keywords:** corneal epithelial cells, neutrophils, particulate matter, inflammation, reactive oxygen

## Abstract

Ocular exposure to particulate matter (PM) causes local inflammation; however, the influence of neutrophils on PM-induced ocular inflammation is still not fully understood. In this study, we constructed a system to investigate the role of PM in ocular inflammation using a co-culture of human corneal epithelial cells (HCE-T) and differentiation-induced neutrophils (dHL-60). To investigate whether HCE-T directly endocytosed PM, we performed a holographic analysis, which showed the endocytosis of PM in HCE-T. The cytokines and chemokines produced by HCE-T were measured using an ELISA. HCE-T treated with PM produced IL-6 and IL-8, which were inhibited by N-Acetyl-L-cysteine (NAC), suggesting the involvement of ROS. Their co-culture with dHL-60 enhanced their production of IL-6, IL-8, and MCP-1. This suggests an inflammatory loop involving intraocular corneal epithelial cells and neutrophils. These cytokines and chemokines are mainly regulated by NF-κB. Therefore, this co-culture system was examined in the presence of an IKK inhibitor known to downregulate NF-κB activity. The IKK inhibitor dramatically suppressed the production of these factors in co-culture supernatants. The results suggest that the inflammatory loop observed in the co-culture is mediated through ROS and the transcription factor NF-κB. Thus, the co-culture system is considered a valuable tool for analyzing complex inflammations.

## 1. Introduction

The cornea is a transparent tissue located at the front of the eyeball; however, since there are no blood vessels in the cornea, there are no hematopoietic immune cells within the corneal tissue unless an injury occurs. Therefore, it is clinically important to understand how the avascular cornea responds to inflammations resulting from injury and infection. When the cornea receives an infection or physical injury, a wound-healing response immediately begins, and inflammatory cells such as neutrophils infiltrate the cornea [1]. While these inflammatory cells play an important role in eliminating microbes [2] and restoring corneal transparency, their persistent presence within the corneal stroma ultimately leads to corneal opacity. During corneal inflammation, inflammatory cytokines are released from cells [3,4].

Neutrophils produce IL-6 and TNF-α after the endocytosis of PM [5]. Neutrophils play an important role in the immune system; however, their short lifespan makes primary cultured neutrophils unsuitable for use in repeated in vitro studies. Treatment with dimethyl sulfoxide (DMSO) or all-trans retinoic acid (ATRA) induces their differentiation into neutrophil-like cells [6,7]. In a previous study, we successfully established a cell line that mimics pro-inflammatory neutrophils using the human cell line HL-60 [8]. We observed that these established cells produce inflammatory cytokines and chemokines such as IL-8 and MCP-1 when they endocytose particles such as PM2.5. In addition to PM2.5, it has also been reported that a similar phenomenon occurs with the endocytosis of Staphylococcus aureus-derived particles (bio-particles) [9]. Although these particles can cause ocular damage, there are no clear studies on the subsequent responses of ocular epithelial cells and neutrophils, and whether these further exacerbate inflammation.

Previous research has demonstrated that the damage to human corneal epithelial cells induced by PM2.5 follows a pattern dependent on both time and dosage [10] and induces significant ROS elevation [11] and corneal toxicity by triggering pyroptosis in human corneal epithelial cells [12]. PM2.5 can induce DNA damage and cell senescence in corneal epithelial cells [13], but there are no in-depth studies on the relationship between PM-induced ocular inflammation and neutrophils.

The transcription factor Nuclear factor-κB (NF-κB) is pivotal in regulating immune responses. The classical form of NF-κB within cells is a heterodimer composed of a p50 subunit and a p65 subunit. It undergoes rapid activation by IKK [14] in response to diverse stimuli, including pathogens, stress signals, and pro-inflammatory cytokines [15,16]. NF-κB translocates to the nucleus of the cell, where it stimulates the transcription of specific inflammatory factors, including IL-6, IL-8, and MCP-1 [17].

To ethically reduce the usage of animals in experiments, there is a growing emphasis on embracing the 3 Rs, the principles of Refinement, Reduction, and Replacement [18,19]. An in vitro experimental system employing corneal epithelial cell lines has emerged as a valuable tool for understanding ocular events [20]. It is important to recognize that although these models are valuable tools, they are currently unable to completely replace animal experiments. Since we have previously reported the establishment of inflammatory-induced neutrophils, we wondered whether it would be possible to construct a new inflammatory system in the eye using differentiating neutrophils and a corneal epithelial cell line.

In this study, we constructed a system to investigate ocular inflammation and the role of PM using a co-culture of human corneal epithelial cells (HCE-T) and differentiation-induced neutrophils (dHL-60).

## 2. Materials and Methods

### 2.1. Materials and Cells

Fluorescent particulate matter (PM, sicastar^®^-redF, particles of different sizes (0.1, 0.3, and 1 μm)) was purchased from Corefront (Waltham, MA, USA). Biological particulate matter was obtained from pHrodo™ Red. Staphylococcus aureus Bioparticles™ Conjugate (BioPM), for phagocytosis (A10010), was purchased from Invitrogen (Carlsbad, CA, USA). The IKK Inhibitor VII (401486) was purchased from Calbiochem (Darmstadt, Germany), and N-Acetyl-L-cysteine (A8199) was purchased from SIGMA-ALDRICH (St. Louis, MI, USA). These inhibitors were dissolved in dimethyl sulfoxide (DMSO) and diluted in PBS before treatment. HCE-T and HL-60 cells were purchased from RIKEN BioResource Center (Tsukuba, Japan).

### 2.2. Cell Cultures, Cells’ Differentiation into Neutrophils, and Flow Cytometry

HCE-T was incubated in Roswell Park Memorial Institute (RPMI) 1640 medium (Nissui, Tokyo, Japan) including 10% fetal bovine serum (Serana Europe GmbH, Brandenburg, Germany), L-glutamine (2 mM, Wako Pure Chemical Industries, Tokyo, Japan), and a penicillin–streptomycin solution (Gibco, New York, NY, USA) and maintained in a humidified incubator with 5% CO_2_ at 37 °C. HCE-T (3 × 10^4^ cells/well/200 µL, in a 48-well plate in Figure 2 and 3 × 10^4^ cells/well/500 µL in a 24-well plate for the co-culture and ROS assay system) were spread. HL-60 cells were differentiated into neutrophils as previously described [8]. Briefly, HL-60 cells were plated in a 6-well plate at a density of 5 × 10^5^ cells/well/2 mL with 1.3% (*v*/*v*) dimethyl sulfoxide (DMSO, Nacalai Tesque, Kyoto, Japan) or 1 µM all-trans retinoic acid (ATRA, R2625, Sigma-Aldrich, St. Louis, MO, USA). The cell differentiation conditions were renewed after 3 days of their 6-day differentiation period. To identify whether HL-60 differentiated into neutrophil-like cells (CD11b-positive), surface markers were analyzed by flow cytometry. The cells were stained with APC-conjugated anti-CD11b (20-0112-U100, Cytek, Fremont, CA, USA) and PerCP Cy5.5-conjugated anti-human CD14 (325622, Biolegend, San Diego, CA, USA) antibodies and incubated for 30 min at 4 °C. The cells were washed and analyzed using a CytoFLEX flow cytometer (Beckman Coulter, Brea, CA, USA). Representative data are shown in Supplementary Figure S1.

### 2.3. Holotomography

HCE-T were treated with PM (1 μm) for 24 h in a specific dish (central glass-bottom TomoDish, Tomocube, 901002-02, Sinseong-ro, Yuseong-gu, Daejeon, 34109, Republic of Korea). Three-dimensional holotomographic images, based on the refractive indexes of the materials, were analyzed following the manufacturer’s instructions using a 3D optical diffraction tomography (3D-ODT) device. Digital staining, based on RI and 3D images, was reconstructed.

### 2.4. Enzyme-Linked Immunosorbent Assay (ELISA)

Enzyme-linked immunosorbent assay kits for human IL-1β, IL-6, IL-8, MCP-1, and TNF-α were purchased from BioLegend (San Diego, CA, USA). Cells were treated with PM (20 μg/mL) or LPS (1 μg/mL) for either 24 or 48 h, with or without inhibitors for 24 h. After treatment, culture supernatants were collected and analyzed for their cytokine levels using a previously described method [21].

### 2.5. Co-Culture HCE-T with HL-60 Using a Transwell

After HCE-T (3 × 10^4^) were adhered to a 24-well plate, HCE-T were co-cultured with DMSO or ATRA differentiated-HL-60 (DHL-60, AHL-60, respectively), or original HL-60 (3 × 10^4^ or 10 × 10^4^) in the presence of PBS, LPS (1 µg/mL), or BioPM (20 µg/mL) for 24 h. The cell culture supernatant was used for the ELISA.

To separate HCE-T and HL-60 cells, a Transwell chamber (Transwell porous cell culture insert, 6.4 mm membrane diameter, 3 μm pore size, polyethylene terephthalate membrane, Corning Caster Corp [Corning, NY, USA]) was used. HCE-T (3 × 10^4^) were seeded into a 24-well plate. The medium was then replaced, with/without DHL-60, and treated with PBS or BioPM while within the Transwell chamber. After 24 h of incubation, supernatants from both the 24-well plate and the Transwell chamber were collected. The combined supernatants from both compartments were used for the ELISA analysis. Figures 3 and 6 use the same experimental setup, with the only difference being the presence of the Transwell chamber.

### 2.6. Measurement of ROS Production

HCE-T (3 × 10^4^) were treated with PBS or NAC (10 mM). After 24 h, the cells were stained with Oxivision Green and observed via a fluorescence microscope. The area of the green fluorescent region was calculated and normalized by the number of cells confirmed in the bright field.

DCFH was used for the measurement of ROS production following the manufacturer’s protocol. HCE-T (3 × 10^4^) were stained with photo-oxidation-resistant DCFH-DA working solution and incubated for 30 min, and fluorescence signals were measured using a plate reader.

### 2.7. Western Blotting

Cells were lysed using RIPA buffer to obtain whole-cell extracts [22]. Equal amounts of protein (10 μg) were separated by electrophoresis. The density of each band was measured using ImageJ software (bundled with 64-bit Java 1.8.0_112; National Institute of Health, Bethesda, MD, USA). The expression levels of target proteins were standardized against β-actin.

### 2.8. Reporter Assay

HCE-T (3 × 10^4^) were transfected using Lipofectamine 2000 Reagent (Invitrogen, Carlsbad, CA, USA) at a ratio of 3 μL of reagent per microgram of DNA, following previously established protocols [15]. In brief, the NF-κB luciferase reporter construct was transfected into cells cultured in 24-well plates at 90% confluency. Later, 24 h post-transfection, the cells were treated with either LPS or an IKK inhibitor for a duration of 6 h. Following stimulation, cell lysates were prepared using Passive Lysis Buffer (Promega, Madison, WI, USA), and their luciferase activity was quantified using a luminometer (Fluoroskan™ FLMicroplate Fluorometer and Luminometer, Thermo Scientific, Waltham, MA, USA).

### 2.9. Ultraviolet (UV) Exposure

HCE-T (3 × 10^4^) were seeded into 24-well plate and incubated for 24 h. The medium RPMI was replaced with PBS. The cells were then exposed to UV-A for 0.5 h (2.6 J/s/m^2^). Then, the PBS was replaced with RPMI medium. After 24 h, culture supernatants were harvested and their IL-6 production was measured using an ELISA kit (Biolegend).

### 2.10. Statistics

The results are presented as mean ± standard deviation. Each column represents the mean level for its respective group. Statistical analyses were conducted using Fisher’s least significant difference (LSD) test following a one-way analysis of variance. Statistical significance was defined as *p* < 0.05. A *t*-test was performed, as seen in Figures 4B and 5B.

## 3. Results

### 3.1. HCE-T’s Ability to Take Up PM Is Analyzed Using Holotomography Technology

**Figure 1:**
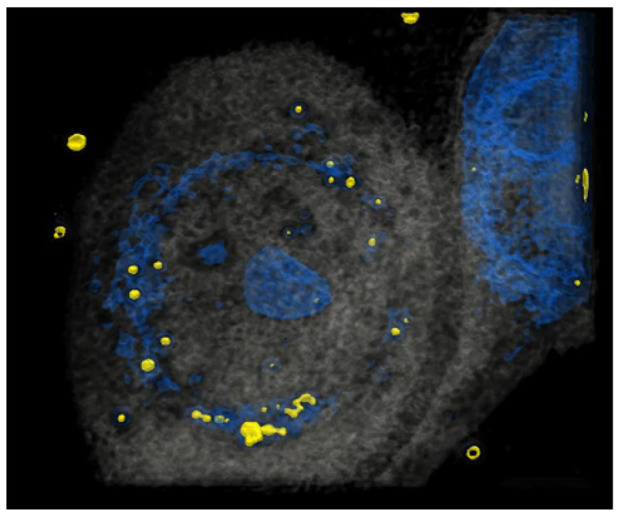
HCE-T takes up PM. At 3 h after HCE-T’s treatment with PM (1 μm), a 3D optical diffraction tomography (3D-ODT) device was employed to capture 3D images of the PM endocytosed by HCE-T. Yellow: 1 μm PM, Blue: nucleus.

It has been reported that HCE-T can take up particles [23]. To investigate precisely whether HCE-T can take up particles in our system, HCE-T were treated with particles, and then a tomography analysis was performed. After the photo was taken, it was reconstructed so that the particles were represented in yellow and the nucleus represented in blue, showing that HCE-T could endocytose particles and exist around the nucleus (Figure 1). We have also included a 3D movie (Supplementary Video S1) in the Supplementary Materials.

### 3.2. HCE-T Produces IL-6 and IL-8 Due to PM Treatment

**Figure 2:**
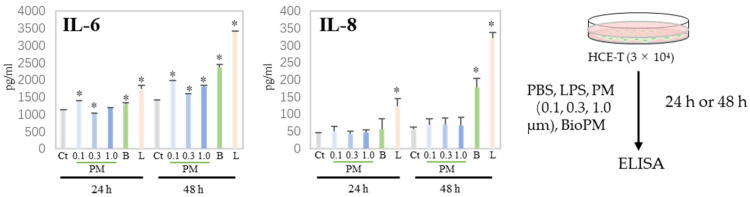
HCE-T produce IL-6 and IL-8 upon PM treatment. HCE-T (3 × 10^4^) were stimulated with PBS; LPS (1 µg/mL); 0.1, 0.3, and 1.0 µm beads (20 µg/mL); and BioPM (20 µg/mL) for 24 h and 48 h. IL-6 and IL-8 production was measured using ELISA. Representative analyses from 3 independent experiments are shown. Ct, control; B, bioparticulate matter (BioPM); L, LPS. * *p* < 0.05 vs. each time point Ct.

Generally, cells produce cytokines during the endocytosis of PM, triggering or enhancing an inflammatory response. We have previously reported that neutrophils have a preferable particle size for endocytosis [5]. Next, H-CET were treated with several particles and the cytokine levels in the supernatant were measured. As shown in Figure 2, IL-6 was induced by several PMs (0.1, 0.3, and BioPM) at 24 h, but not IL-8. However, IL-8 was detected in BioPM-treated cells at 48 h. In contrast, the IL-1ꞵ, TNF-α, and MCP-1 levels were below the detection level. These results suggest that HCE-T endocytose PM and produce inflammatory factors.

### 3.3. Co-Culture with Differentiated HL-60 Cells Enhances the IL-6, IL-8, and MCP-1 Production in HCE-T

**Figure 3:**
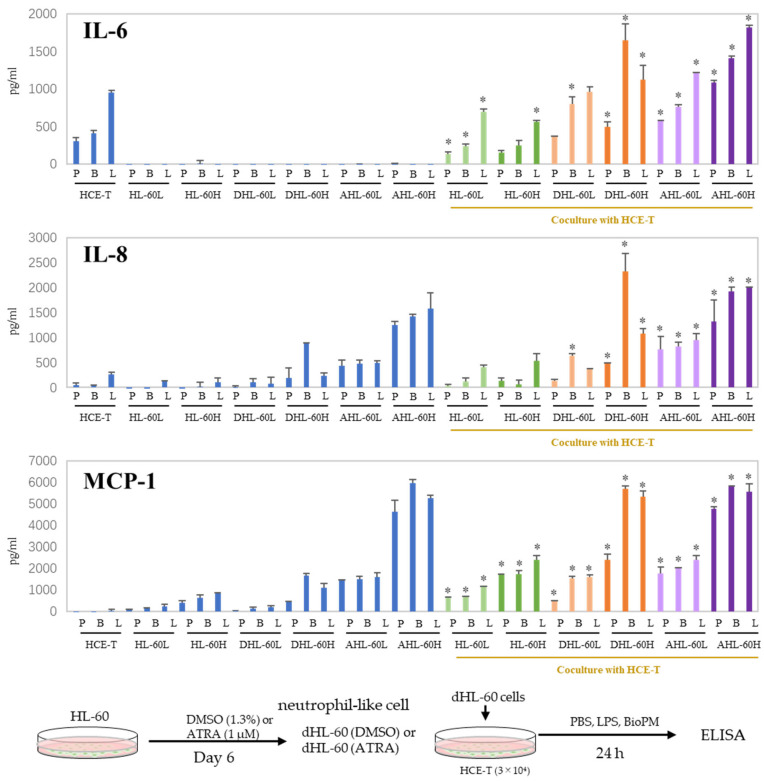
Co-culture with HL-60 enhances IL-6, IL-8, and MCP-1 production in HCE-T. HL-60 was differentiated into neutrophils by DMSO (1.3%) and ATRA (1 µM) over 6 days. HCE-T (3 × 10^4^) were co-cultured with DMSO- or ATRA-differentiated and original HL-60 (3 × 10^4^ or 10 × 10^4^) in the presence of PBS (P), LPS (L, 1 µg/mL), or BioPM (B, 20 µg/mL) for 24 h. Cytokine and chemokine levels were analyzed using ELISA. Representative analyses from 3 independent experiments are shown. DHL-60: DMSO-differentiated HL-60, AHL-60: ATRA-differentiated HL-60. (HL-60L: 3 × 10^4^, HL-60H: 10 × 10^4^). Blue column: single culture, green column: co-culture with undifferentiated HL-60, orange column: co-culture with DMSO-differentiated HL-60, purple column: co-culture with ATRA-differentiated HL-60. * *p* < 0.05 vs. each comparable HCE-T treatment.

When inflammation arises, neutrophils are recruited to the site of inflammation. In our previous study, we demonstrated that HL-60 cells can be differentiated into neutrophil-like cells by DMSO and ATRA. DHL-60, AHL-60, undifferentiated HL-60, and HCE-T were co-cultured to investigate their effects on recruited neutrophils. IL-6 and IL-8 production was further increased compared to that of mono-cultured cells (Figure 3). No production of TNF-α, IL-1β, or IL-17A was observed even after co-culturing. On the other hand, an enhanced production of MCP-1 was observed in the co-cultures. These results imply that neutrophils can amplify the inflammatory response in the cornea. In the following experiments, DMSO-differentiated HL-60 is used for the co-culture system because we reported that DMSO-induced neutrophils more closely mimic the properties and data of murine proinflammatory neutrophils than ATRA-induced ones [8], and their response to BioPM is higher than that of ATRA-differentiated HL-60.

### 3.4. ROS Are Involved in the Production of Cytokines and Chemokines in Co-Culture Systems

**Figure 4:**
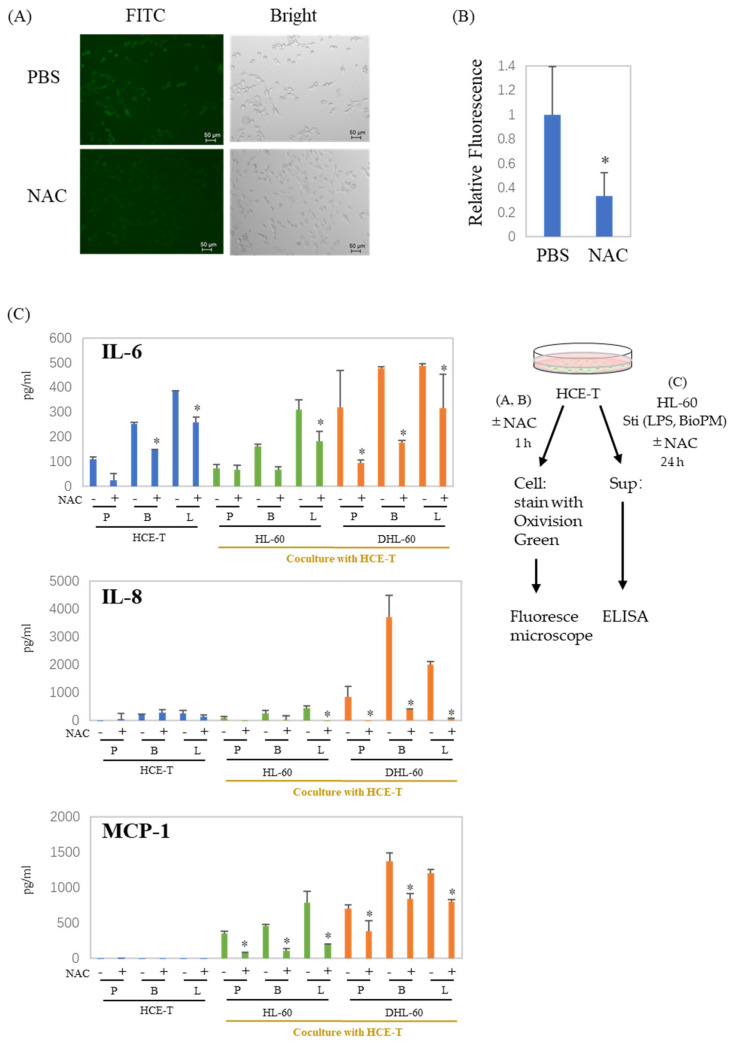
ROS are involved in the production of cytokines and chemokines in co-culture systems. (**A**,**B**) HCE-T (1 × 10^4^) were treated with PBS or NAC (10 mM) for 1 h, and cells were stained with Oxivision Green and analyzed by fluoresce microscope. A representative photo is shown in (**A**). (**B**) The area of the green fluorescent region was calculated and normalized by the number of cells confirmed in the bright field. The graph shows the average ± SD of the four regions. The PBS group is represented as 1. * *p* < 0.05, vs. PBS. (**C**) HCE-T (3 × 10^4^) were co-cultured with DMSO-differentiated and original HL-60 cells (10 × 10^4^) in the presence of PBS (P), LPS (L, 1 µg/mL), or BioPM (B, 20 µg/mL), with or without NAC (10 mM). IL-6, IL-8, and MCP-1 levels were analyzed by ELISA. Representative analyses from 2 independent experiments are shown. DHL-60: DMSO-differentiated HL-60 cells. * *p* < 0.05 vs. the without-NAC sample of each group.

NAC is widely used as a scavenger of ROS production. To examine whether ROS are associated with the production of enhanced soluble factors, NAC was applied to our co-culture system. Surprisingly, HCE-T continuously produce ROS (Figure 4A, green fluoresce cells), which are dramatically suppressed by NAC (Figure 4A,B). As expected, there was a significant decrease in the production of IL-6, IL-8, and MCP-1 in the presence of NAC (Figure 4C). These results indicate that ROS are involved in the production of inflammatory cytokines and chemokines, regardless of whether they are produced by a co-culture or not. This suggests a connection between the inflammation observed in our system and ROS.

### 3.5. IL-6, IL-8, and MCP-1 Production Are Inhibited by an IKK Inhibitor after HCE-T Are Co-Cultured with Neutrophil-like Cells

**Figure 5:**
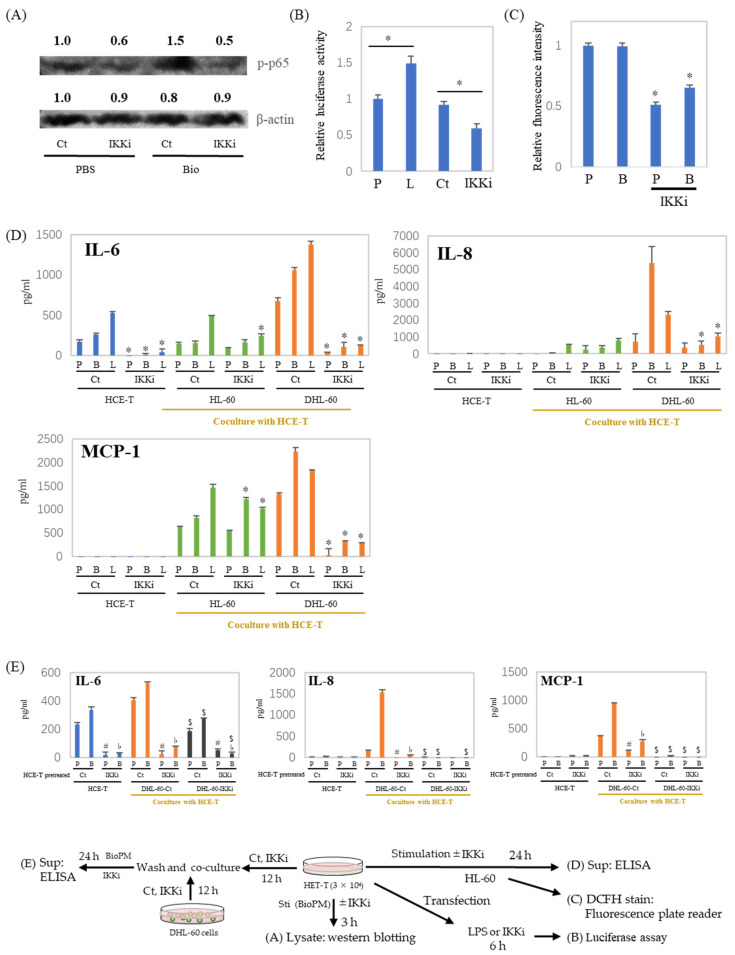
IL-6, IL-8, and MCP-1 production in a co-culture with neutrophil-like cells is inhibited by an IKK inhibitor. (**A**) HCE-T (3 × 10^4^) were treated with BioPM, in the presence or absence of IKK Inhibitor (IKKi, 10 μM), for 3 h. Cell lysate was used for Westeren blotting. Phosphorylated p65 at Serine 536 (p-p65) was detected by immunoblotting, with beta-actin serving as the loading control. The relative density of the bands is indicated by the numbers above each band. The densities from the control band (PBS treatment) were utilized as the reference (1.0) to determine the relative band intensities. (**B**) HCE-T (3 × 10^4^) were transfected using a luciferase reporter construct. Cells were treated with LPS or IKK inhibitor 24 h after transfection for 6 h and cell lysates were used for luciferase assay. (**C**,**D**) HCE-T (3 × 10^4^) were co-cultured with DMSO-differentiated or original HL-60 (10 × 10^4^) in the presence of PBS (P), BioPM (B, 20 µg/mL), or LPS (L, 1 µg/mL) with or without IKK Inhibitor (IKKi, 10 μM) for 24 h, DHL-60 were harvested and ROS production was assessed by DCFH stain (**C**), the supernatants were used for ELISA (**D**). (**E**) HCE-T (3 × 10^4^) were pre-treated with the IKK inhibitor (IKKi, 10 μM) for 12 h and co-cultured with DHL-60 (Ct) or IKKi pre-treated DHL-60 (10 × 10^4^) for 24 h. Cytokine levels were analyzed by ELISA. DHL-Ct: DHL-60 pre-treatment with control for 12 h before co-culture, DHL-IKKi: DHL-60 pre-treatment with IKKi for 12 h before co-culture. Ct, control (0.1% DMSO), DHL-60H: DMSO-differentiated HL-60. Representative analyses from 2 independent experiments are shown. * *p* < 0.05 vs. Ct of each group. ^#^ *p* < 0.05, pretreated HCE-T Ct vs. IKKi, PBS stimulation. ^♭^ *p* < 0.05, pretreated HCE-T Ct vs. IKKi, BioPM stimulation. ^$^ *p* < 0.05, DHL-60 pretreated Ct vs. IKKi.

NF-κB is a pivotal regulator of inflammatory cytokine production. Our previous investigations have demonstrated that the inhibition of IKK suppresses endocytosis [9]. To investigate the role of NF-κB in inflammatory cytokine production, an IKK inhibitor was applied to the co-culture system. As shown in Figure 5A, the IKK inhibitor inhibits p65 phosphorylation. In addition, transcription activity is also inhibited by the IKK inhibitor (Figure 5B). ELISA data show a dramatic reduction in IL-6 production in HCE-T at both 24 h (Figure 5D, blue column) and 48 h (Supplementary Figure S2A). This was also demonstrated by the fact that other NF-κB inhibitors similarly inhibited stimulus-induced IL-6 production (Supplementary Figure S2B). Furthermore, the ROS production of DHL-60 was also attenuated by the IKK inhibitor (Figure 5C). In addition, the enhanced production of inflammatory cytokines and chemokines in the co-culture system, in the presence or absence of BioPM, was also suppressed by the IKK inhibitor. (Figure 5D, green and orange column). To investigate the cells contributing to enhanced cytokine production in the co-cultures, DHL-60 were pretreated with an IKK inhibitor and co-cultured. When DHL-60 were pretreated with an IKK inhibitor, their IL-6, IL-8, and MCP-1 production in response to BioPM were dramatically reduced in co-culture (Figure 5E, black column). Additionally, when HCE-T were pre-treated solely with an IKK inhibitor before co-culturing, there was a notable decrease in their production of IL-8 and MCP-1, even with the subsequent absence of the inhibitor (Figure 5E).

### 3.6. Cell–Cell Interactions Affect Cytokines and Chemokines’ Production

**Figure 6:**
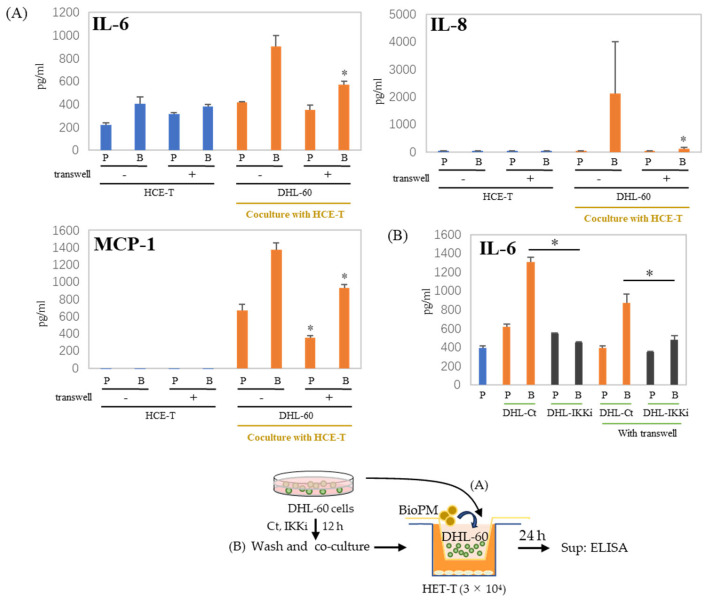
Production of cytokines and chemokines was decreased by the insertion of a transwell. (**A**) HCE-T (3 × 10^4^) were co-cultured with DHL-60 (10 × 10^4^) in the presence of PBS (P) or BioPM (B, 20 µg/mL), with or without a transwell. (**B**) HCE-T (3 × 10^4^) were co-cultured with pre-treated DHL-60, in the presence or absence of a transwell. * *p* < 0.05 vs. Bio-treated Ct. Cytokine and chemokine levels were analyzed by ELISA. Representative analyses from 2 independent experiments are shown. DHL-60: DMSO-differentiated HL-60. DHL-Ct: DHL-60 pre-treatment with control for 12 h before co-culture, DHL-IKKi: DHL-60 pre-treatment with IKKi for 12 h before co-culture. Ct, control (0.1% DMSO). * *p* < 0.05, vs. without a transwell.

In the co-culture of HCE-T with HL-60, there are cell–cell interactions. We used a transwell to separate HCE-T and HL-60 to demonstrate whether this cell–cell interaction affects our co-culture system. There is reduction after the transwell is used, as shown in the results of IL-6 and MCP-1 production (Figure 6A). This decrease in IL-6 production was more effectively suppressed when DHL-60 was pretreated with an IKK inhibitor (Figure 6B). IL-8 and MCP-1 production were completely inhibited by pre-treatment with IKKi of DHL-60 (Supplementary Figure S3). Therefore, cell–cell interactions can be important in the production of cytokines and chemokines in co-culture systems.

## 4. Discussion

**Figure 7:**
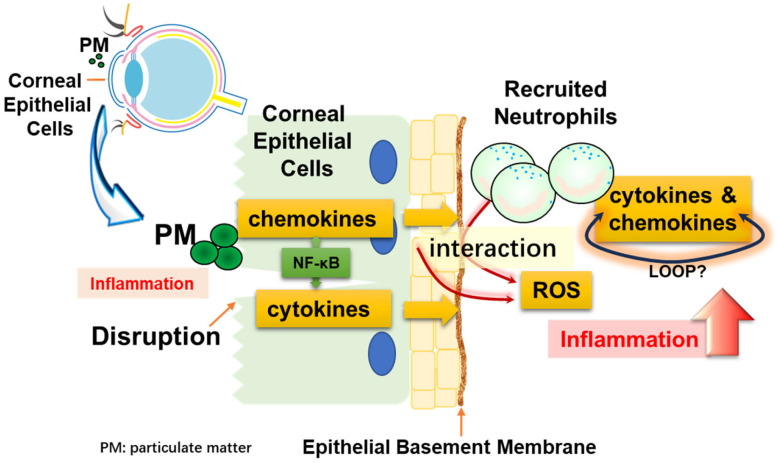
Diagrammatic representation. Corneal epithelial cells may be damaged by physical stimulation. These cells can directly take up particles (PM), resulting in inflammation. At this time, blood vessels are regenerated and neutrophils, which are immune cells, are recruited. Inflammation is exacerbated by the interaction of the recruited neutrophils with particulate matter, and cell–cell interactions occur between corneal epithelial cells and neutrophils.

In this study, we proposed a cell line system that mimics the situation in which corneal epithelial cells are damaged by particulate matter and immune cells are recruited. We demonstrated that when both cells endocytose particles, they produce cytokines and chemokines that indicate inflammation. Furthermore, we showed that recruited neutrophils significantly amplified the inflammatory response through NF-κB and ROS. Figure 7 shows these interactions as a simple illustration.

The air pollution caused by PM2.5 is a serious concern in numerous Asian countries [24]. PM2.5, a critical element of atmospheric pollution, can attack a variety of different organs in the body [25]. In particular, eye epithelial cells that are subject to direct airborne contact with pollutants may become more susceptible to inflammation [26,27]. As we previously showed that PM induces inflammation [28], PM was also thought to be involved in inducing inflammation in the cornea. Nagai et al. showed that HCE-T can uptake nanoparticle beads, suggesting that PM2.5 can be phagocytized into HCE-T and damage cells [29]. Holotomography technology provides label-free 4D quantitative imaging solutions for imaging and cell analyses. Importantly, it allows for subcellular organelles to be observed in live cells without fixation, transfection, or antibody staining [30]. In this study, we demonstrated that HCE-T can take up 1 µm diameter PE-conjugated beads without staining them, using holotomography technology. To our knowledge, this is the first direct evidence that HCE-T take up PM without any treatment being used for the analysis.

In this study, we present a system that mimics the situation in which immune-competent cells flow into the cornea after an injury occurs in the cornea, which lacks a vascular system. We showed that corneal epithelial cells alone induce inflammation in response to PM, but when neutrophils influx or exist there, inflammation is synergistically exacerbated. At this time, considering that the effect was limited only by adding cells that had not yet differentiated into neutrophils, it is expected that the inflammatory cells that flowed in made a large contribution to this inflammatory response. In particular, the dramatic change in chemokine production (IL-8) in the co-culture system suggests an exacerbated inflammatory loop caused by cells that subsequently migrate further.

Consistent with previous reports [31], we demonstrated that ROS are involved in cytokine and chemokine production in this co-culture system. PM 2.5 can induce DNA damage in corneal epithelial cells, probably by promoting ROS formation [13]. IL-6, ROS, NF-κB, and UV are closely related, and UV irradiation can easily occur in the eye. It has been reported that UV-A exposure induces mitochondrial damage, ROS production, and NF-κB activation in HCE-T, and decreases the cell barrier function [32,33]. In addition, Benko et al. demonstrated that UV exposure increases IL-6 production [34]. We also briefly examined the cellular response to UV exposure. Our results suggest that UV exposure increases basal-level IL-6 production from HCE-T (Supplementary Figure S4). Since IL-6 is a senescence biomarker, this suggests that UV induces cell damage or senescence. However, there are still no detailed studies using our system regarding the mechanisms by which HCE-T undergo cellular senescence after UV exposure and the inflammation that is exacerbated by injury. In the future, we hope to use our system to obtain information about these relationships.

Following endocytosis, neutrophils promptly generate inflammatory cytokines [5,35]. The NF-κB signaling pathway primarily influences the production of IL-6 and IL-8 by enhancing the transcriptional activity of these genes [17,36]. In our previous paper, IKK inhibitor VII, which inhibits NF-κB signaling, completely abrogated the production of these cytokines in these cells before their stimulation with PM [5,9]. Therefore, our observations regarding the effects of IKK inhibitors on the co-culture system in this study suggest that IKK inhibitors are probably partially affecting differentiated HL-60. Furthermore, since the phosphorylation of the p65 of NF-κB is inhibited, it is expected that HCE-T are also influenced by IKK inhibitors and affect cytokine production. In other words, the phenomenon of enhanced inflammation observed in this co-culture is thought to be due to the effect of the IKK inhibitor on both cells.

Here, we demonstrated that cell–cell contact was partially responsible for the phenomena observed in this co-culture system. Furthermore, regarding the production of IL-6, we may be able to make some guesses about the producing cells. As shown in Figure 3, DHL-60 does not produce much IL-6, which is consistent with Klein M.B. et al.’s comment [37]. Therefore, the enhanced IL-6 production observed in our co-culture is likely derived from HCE-T. In Figure 6, the presence of a transwell suppresses this enhancement. Interestingly, when HCE-T were pre-treated solely with an IKK inhibitor before co-culture, there was a notable decrease in the production of IL-8 and MCP-1. This indicates that HCE-T also play a role in their augmented production during co-culture, although HL-60 cells seem to be the primary source of these cytokines. This suggests that cell–cell contact influences HCE-T-derived IL-6 production. Furthermore, the antibody neutralization of IL-6 impacts the production of IL-8 and MCP-1 in this co-culture, indicating that an inflammatory loop is involved in this system (Supplementary Figure S5). Even in the AHL-60 co-culture system, IL-6 and IL-8 production tends to be attenuated in transwells.

Although a disruption of immune cells (neutrophils) was not evident under the microscope, it is thought that Neutrophil Extracellular Traps (NETs) [38,39], one of the characteristics of neutrophils, are formed within this system. NETs have been identified as triggers for a self-limited inflammatory reaction [40]. At this time, endogenous contents may be released, which may secondarily affect epithelial cells. In fact, our paper also demonstrated that differentiated neutrophils exerted a NET-like phenomenon [8]. Furthermore, the observed transwell-inhibited experimental data did not display a complete elimination of the effect of the co-culture. In fact, when the co-culture was observed using a fluorescence microscope, the presence of the transwell was seen to reduce cell–cell interactions (Supplementary Figure S6). In the co-culture system, it is expected that neutrophils will phagocytose particles and release soluble factors immediately. These influences may still persist. Additionally, while the pore size of the transwell used in this study was 3 microns, when considering the motility of neutrophils, it may be more effective to use a transwell with a smaller pore size.

Ocular epithelial cells serve as the primary physical barrier against foreign substances. This barrier is susceptible to weakening or breakdown under various influences. Factors such as dry eye and cellular aging contribute to this vulnerability. The system demonstrated in this study is deemed optimal for replicating these phenomena.

## 5. Conclusions

In this study, we investigated how PM causes ocular inflammation and how pro-inflammatory neutrophils are involved in this process. We were able to demonstrate that HCE-T can directly endocytose PM without using antibodies. However, cytokine production, which is a sign of inflammation, was dramatically enhanced by HCE-T’s co-culture with neutrophils, and cell-to-cell contact was important for this inflammatory loop due to the suppressive effect of the transwell on cytokine production. It was also suggested that these inflammatory responses are mediated through ROS and NF-κB signaling pathways. These findings highlight the importance of studying PM-induced ocular inflammation in co-culture systems to understand complex inflammatory mechanisms. The in vitro system using corneal epithelial cells and neutrophils that we have demonstrated here will be of great value in predicting the ocular inflammation caused by various substances in the future.

## Data Availability

The data presented in this study are available upon request from the corresponding author.

## Supplementary Materials

The following supporting information can be downloaded at https://www.mdpi.com/article/10.3390/antiox13040467/s1, Video S1: HCE-T take up PM. Figure S1: HL-60 differentiated into neutrophil-like cells. Figure S2: NF-κB inhibitors inhibit cytokine production. Figure S3: IL-8 and MCP-1 production were inhibited in pre-treated DHL-60 by IKKi. Figure S4: UV-A exposure has effects on the production of IL-6. Figure S5: Cytokines’ production was inhibited by anti-IL-6 antibody in co-culture. Figure S6: Photo from HCE-T co-culture with DHL-60.
