# Supplementary material for: Inflammatory Cytokines and Chemokines Are Synergistically Induced in a ROS-Dependent Manner by a Co-Culture of Corneal Epithelial Cells and Neutrophil-like Cells in the Presence of Particulate Matter
Source: Antioxidants (Basel). 2024 Apr 16;13(4):467. doi: 10.3390/antiox13040467 (PMC11047649; doi:10.3390/antiox13040467)
Supplement: Supplementary file 1 [file antioxidants-13-00467-s001.zip › antioxidants-2917673-supplementary.pdf]

Supplementary Video S1

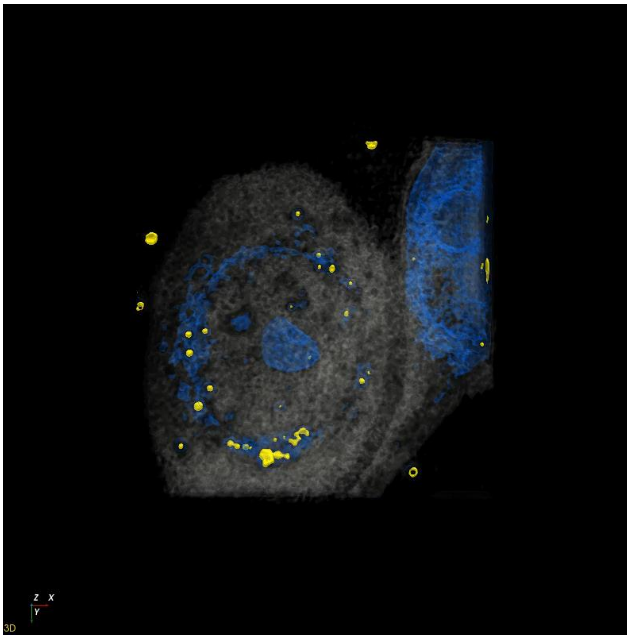

Supplementary Video S1. HCE-T take up PM. At 3 h after HCE-T treatment with PM (1 μm), a 3D optical diffraction tomography (3D-ODT) device was used to capture 3D images of PM endocytosed by HCE-T. Yellow: 1 μm PM, Blue: nucleus.

Supplementary Figure S1

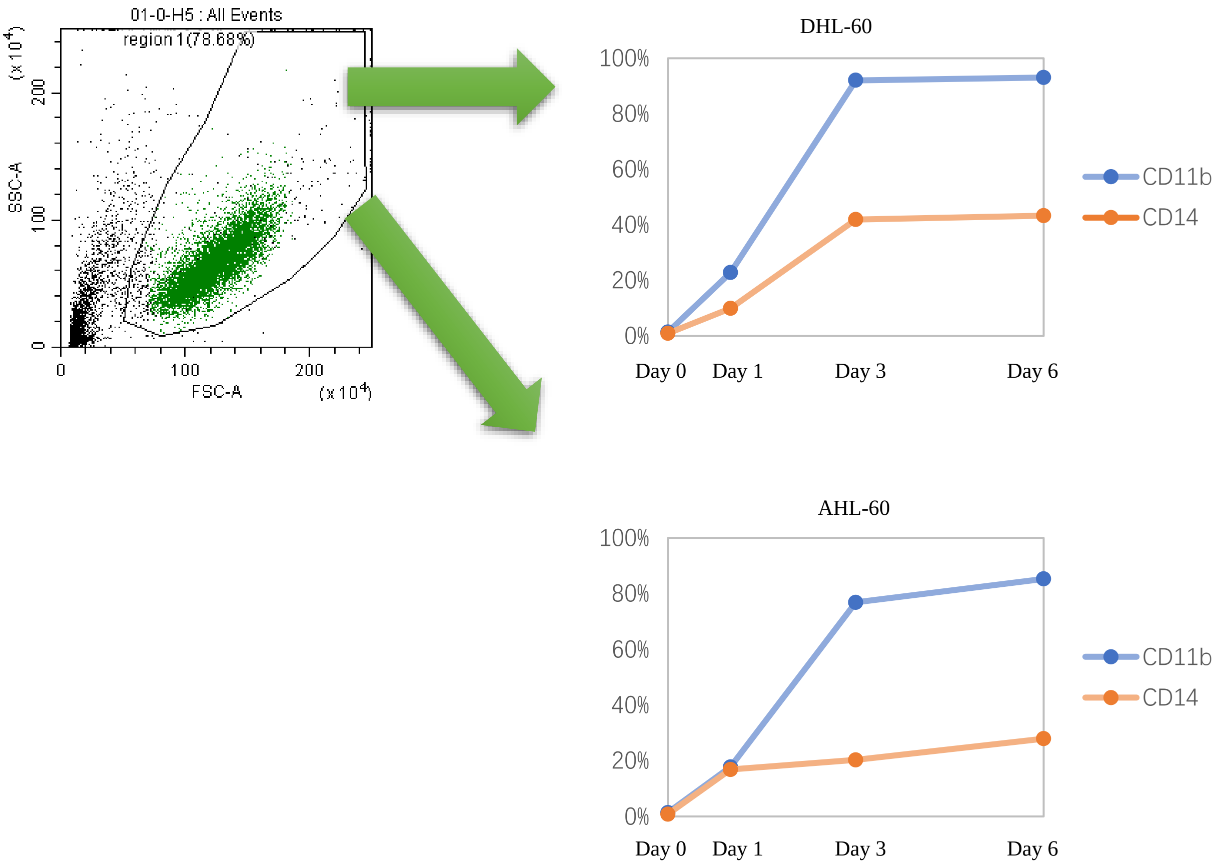

Supplementary Figure S1. HL-60 differentiated into neutrophil-like cells. HL-60 cells were plated in a 6-well plate at a density of 5 × 10^5 cells/well/2 ml of 1.3% (v/v) DMSO or 1 μM ATRA. The cell differentiation conditions were renewed after 3 days for 6-days differentiation period. DMSO- (DHL-60) or ATRA (AHL-60)-differentiated HL-60 cells from indicated days were harvest and stained with APC anti-CD11b antibody or PerCP-Cyanine5.5 anti-CD14 antibody and analyzed by flow cytometry. Original HL-60 (non-differentiated HL-60 cells) data are shown as day 0. The left panel displays scatter plot analysis of SS and FS. The percentage of CD11b and CD14 positive cells from region 1 were showed in DHL-60 and AHL-60 as indicated days.

Supplementary Figure S2

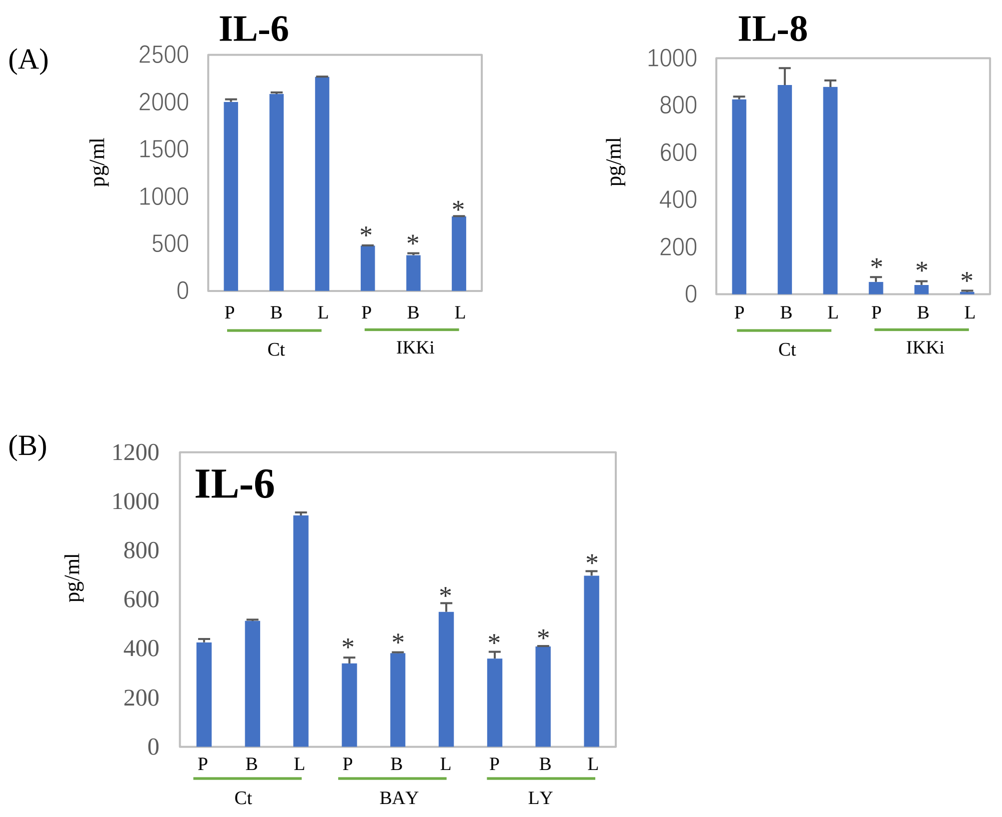

Supplementary Figure S2. NF-κB inhibitors inhibit cytokine production. HCE-T (3×10^4) was stimulated with PBS (P), BioPM (B, 20 μg/ml), and LPS (L, 1 μg/ml) in the presence or absence of IKKi (10 μM), BAY 11-7085 (BAY, 5 μM), and LY294002 (LY, 10 μM) for 48 h (A) or 24 h (B). IL-6 (A, B) and IL-8 (A) in cell culture supernatant were measured by ELISA. Ct, control (0.1% DMSO). *$p < 0.05$ vs. solvent control (Ct). BAY (BML-EI279) was purchased from Enzo Life Sciences (Farmingdale, NY, U.S.A.). LY (#9901S) was purchased from Cell Signaling Technology (Danvers, MA, U.S.A.).

Supplementary Figure S3

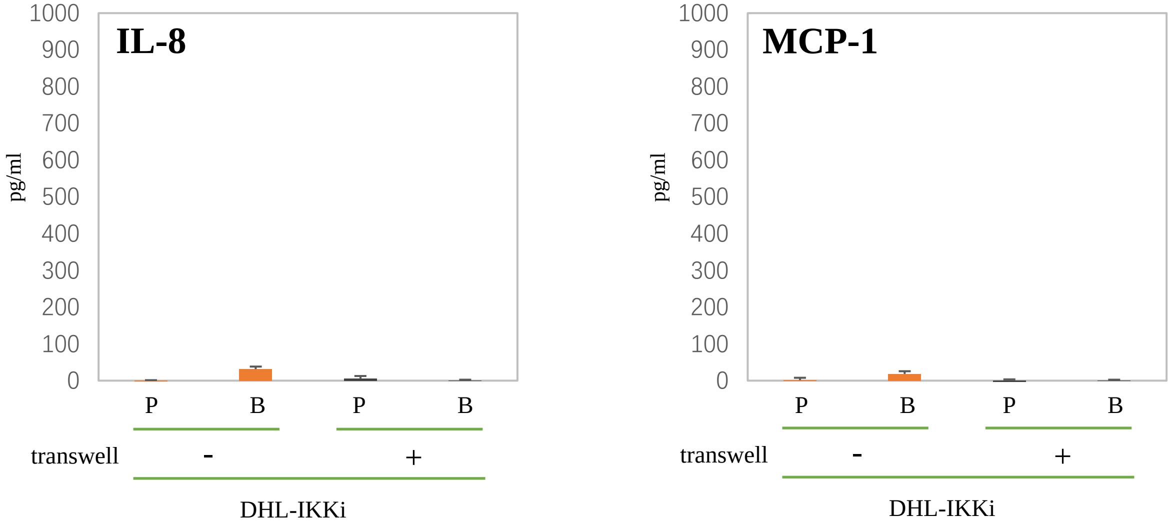

Supplementary Figure S3. IL-8 and MCP-1 production were inhibited in pre-treated DHL-60 by IKKi. HCE-T (3 × 10^4) was co-cultured with IKKi (10 μM) pre-treated DHL-60 in the presence or absence of transwell and stimulated with PBS (P) or BioBM (B, 20 μg/ml) for 24 h. IL-8 and MCP-1 levels were analyzed by ELISA. Representative analyses from 2 independent experiments are shown. DHL-IKKi: DHL-60 pre-treatment with IKKi for 12 h before co-culture.

Supplementary Figure S4

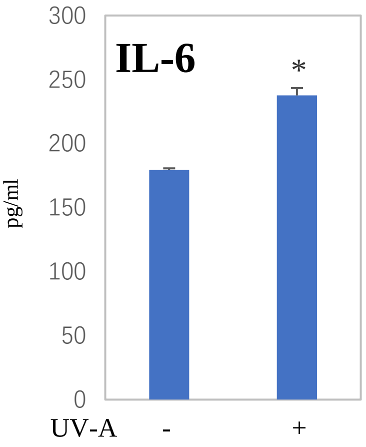

Supplementary Figure S4. UV-A exposure has effects on the production of IL-6. HCE-T (3×10^4) was exposed to UV-A (365 nm wavelength) for 0.5 h. After 24 h of culture, IL-6 production in culture supernatants was measured. *$p < 0.05$ vs. without UV-A. Analysis by *t*-test.

Supplementary Figure S5

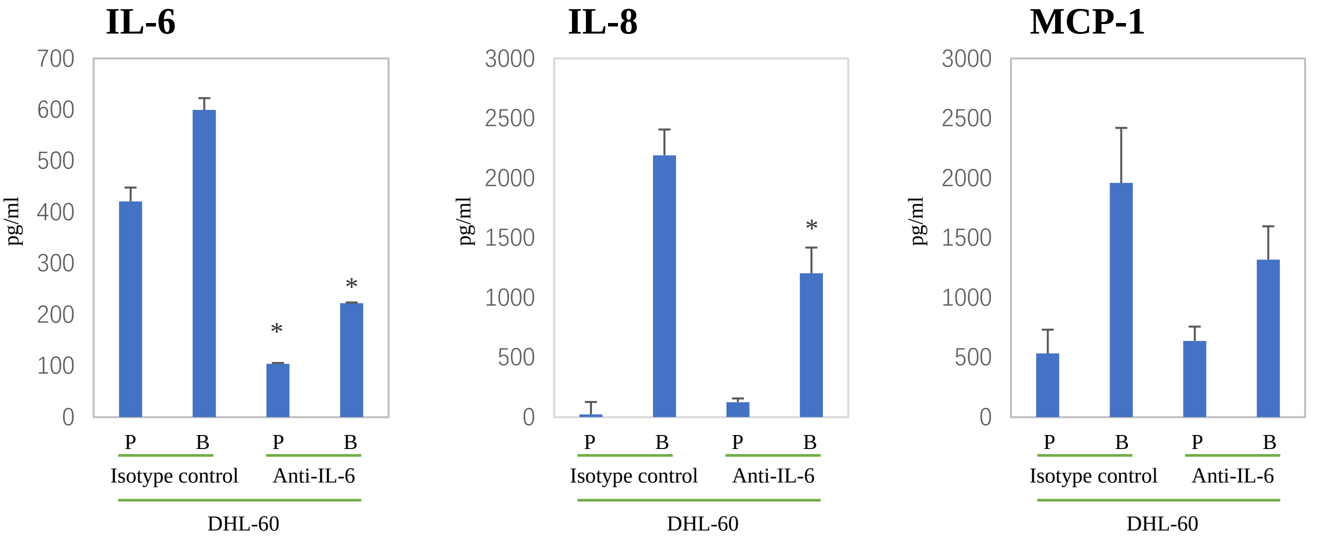

Supplementary Figure S5. Cytokines' production was inhibited by anti-IL-6 antibody in co-culture. HCE-T (3×10^4) was stimulated with PBS (P) and BioPM (B, 20 µg/ml) in the presence or absence of anti-IL-6 antibody or isotype control for neutralization (10 ng/ml), for 24 h. IL-6, IL-8, and MCP-1 levels in cell culture supernatant were measured by ELISA. DHL-60: DMSO-differentiated HL-60 cells. *$p < 0.05$ vs. isotype control. Anti-IL-6 (Human IL-6 Antibody, AF-206-SP) and isotype control was purchased from R&D Systems (614 McKinley Place NE, Minneapolis, MN 55413, U.S.A.).

Supplementary Figure S6

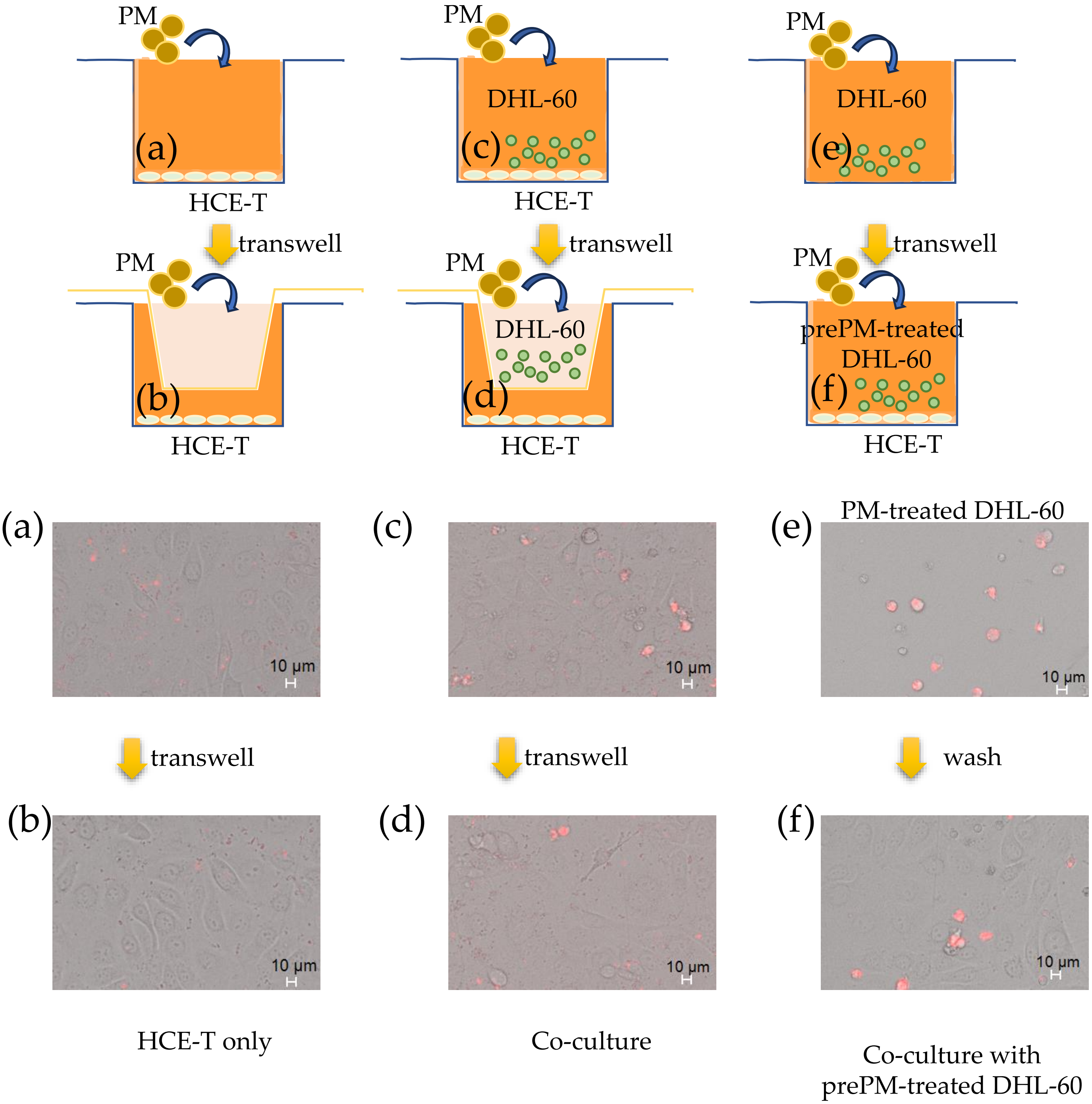

Supplementary Figure S6. Photo from HCE-T co-culture with DHL-60. HCE-T (3×10^4) was co-cultured with DMSO-differentiated HL-60 (10×10^4) in the presence of BioPM (20 μg/ml), with (b, d) or without (a, c, f) transwell. PM: BioPM, DHL-60: DMSO-differentiated HL-60 (10×10^4/well). (e) pretreated DHL-60 with BioPM, (f) HCE-T co-culture with pretreated DHL-60 without transwell. Each photo was taken by fluoresce microscope and representative photos were shown.
